# Ectrodactyly, Ectodermal Dysplasia, Cleft Lip, and Palate (EEC Syndrome) with Tetralogy of Fallot: A Very Rare Combination

**DOI:** 10.3389/fped.2015.00051

**Published:** 2015-06-16

**Authors:** Deepak Sharma, Chetan Kumar, Sanjay Bhalerao, Aakash Pandita, Sweta Shastri, Pradeep Sharma

**Affiliations:** ^1^Department of Neonatology, Fernandez Hospital, Hyderabad, India; ^2^Department of Pediatrics, Madras Institute of Orthopaedics and Traumatology, Chennai, India; ^3^ACPM Medical College, Dhule, India; ^4^Rabindranath Tagore Medical College, Udaipur, India

**Keywords:** ectrodactyly ectodermal dysplasia–cleft, cleft hand or lobster claw hand/foot, Tetralogy of Fallot, TP63 gene, R280C mutation

## Abstract

Ectrodactyly, ectodermal dysplasia, and cleft lip/palate syndrome (EEC) syndrome is a rare genetic disorder with an incidence of around 1 in 90,000 in population. It is known with various names including split hand–split foot–ectodermal dysplasia–cleft syndrome or split hand, cleft hand, or lobster claw hand/foot. We report first case of EEC with associated heart disease (Tetralogy of Fallot) who was diagnosed as EEC on the basis of clinical features and EEC was confirmed with genetic analysis.

## Introduction

Ectrodactyly ectodermal dysplasia-cleft (EEC) syndrome is an autosomal dominant disorder characterized by the triad of ectrodactyly (development of anomalies of the structures derived from the embryonic ectodermal layer), ectrodactyly (extremities, hands and feet malformations), and cleft lip and/or palate. These malformations can be seen all together in a neonate or in isolation. EEC usually is not associated with congenital heart disease and has been reported rarely in medical literature. We report a male child who was diagnosed as a case of EEC with associated heart disease (Tetralogy of Fallot TOF). This is the first case report to the best of our knowledge reporting EEC with TOF.

## Case Presentation

**Figure 1:**
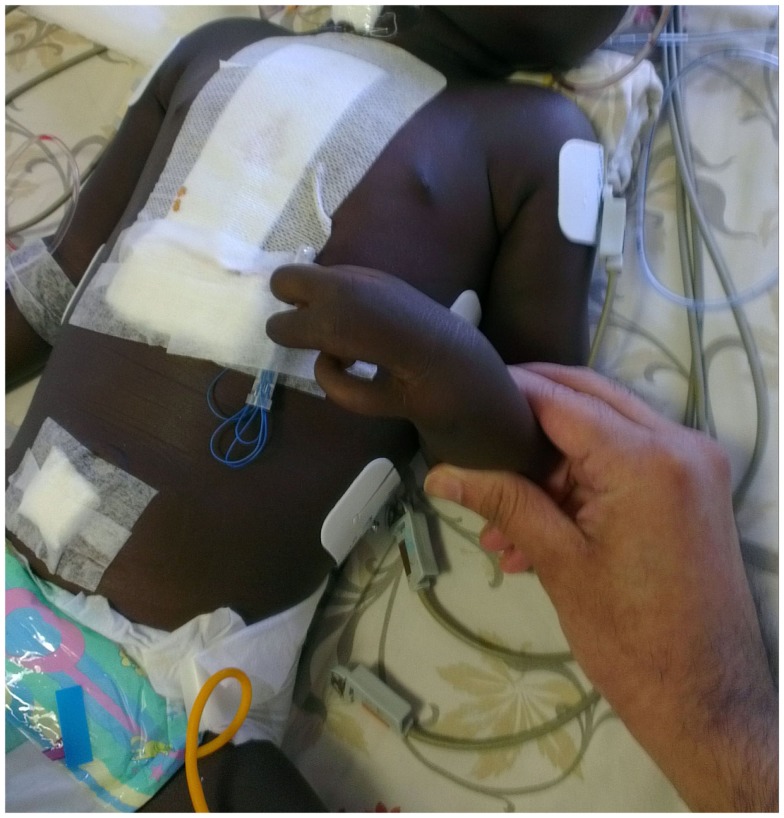
**Figure showing left hand with fusion of middle and ring finger giving the appearance of lobster hand**.

This 2½-year-old male child was referred to our hospital for corrective surgery of Tetralogy of Fallot. The index case was first child born to a non-consanguineous couple. The child presented with complaints of intermittent episodes of bluish discoloration of lips since age of 1 year and at the age of 2, he was evaluated by a local doctor and was diagnosed to have TOF and hence was referred here for further management. On physical examination, the child was noted to be cyanosed, with presence of ectrodactyly in both hands (Figure 1). There was no family history of EEC or other genetic abnormality in the family. The child was also noted to have scaling of skin and poor dentition clinically. Systemic examination revealed precordial bulge with a grade 2/6 ejection systolic murmur in the left sternal border. On retrospective enquiry the parents informed us that the child also had decreased sweating and on–off febrile episodes, the child was evaluated for the skin condition in their hometown although the details were not available. Echocardiography revealed TOF for which the baby underwent corrective surgery. The provisional diagnosis of EEC syndrome with TOF was kept on the basis of claw-like hand and other clinical features and the infant was evaluated with genetic analysis of EEC syndrome. The genetic analysis of the patient showed mutation inTP63gene (R280Cmutation) causing EEC type 4. A dermatologist consult was sought who advised for a skin biopsy as the parents were not willing for the skin biopsy it was with-held. The child’s thyroid screen as well as hearing screen done was normal. The child was discharged but was lost in follow up. The consent was taken from parents for case report publication.

## Discussion

Ectrodactyly, ectodermal dysplasia, and cleft lip/palate syndrome syndrome is a rare genetic disorder with an incidence of around 1 in 90,000 in general population^1^. It is known with various names that includes split hand–split foot–ectodermal dysplasia–cleft syndrome or split hand, cleft hand, or lobster claw hand/foot (1). It has been postulated to be caused by mutation in TP63 gene. EEC syndrome is usually inherited as an autosomal dominant trait although sporadic cases have also been reported. TP63 gene is located on the long arm (q) of chromosome 3 (3q27). TP63 encodes a homolog of the tumor suppressor p53 gene (2). TP63 has been found to have around six isoforms, with their prime function being modulating gene expression (3). There are other four syndromes reported in medical literature that are caused by mutations of the p63 gene including Ankyloblepharon-Ectodermal dysplasia-Clefting (AEC syndrome, MIM 106260), AcroDermato-Ungueal-Lacrimal-Tooth (ADULT syndrome, MIM 103285), Rapp–Hodgkin (RHS syndrome, MIM 129400), and Limb-Mammary (LMS syndrome, MIM 603543) (Table 1).There is considerable overlap among these disorders and some researchers have postulated them as different spectrum of same disorder due to differential expression of genes. There has been rare situation in which patients affected with EEC syndrome are noted to have chromosomal deletions or translocations on the long arm of chromosome 7 (7q11.2–q21.3 and 9p12) (4, 5).

**Table 1:** **Different overlapping diseases with EEC and their main features are shown**.

Disease	Clinical features
Ankyloblepharon-ectodermal dysplasia-clefting (AEC syndrome, MIM 106260)	Characterized by ankyloblepharon (congenital adhesions of the eyelids), ectodermal dysplasia, brittle white, and sparse eyebrows and eyelashes, otitis media, nevi, and orofacial clefts (6, 7)
Acrodermato-ungueal-lacrimal-tooth (ADULT syndrome, MIM 103285)	Characterized by ectrodactyly, syndactyly, excessive freckling, dry skin, dysplastic nails, lacrimal duct atresia, primary hypodontia, and early loss of permanent teeth (8, 9)
Rapp–Hodgkin (RHS syndrome, MIM 129400)	Characterized by cleft lip and palate, small mouth, narrow nose, coarse and wiry hairs progressing to alopecia in adults, oligodontia or anodontia, hypoplasia of the nails, abnormalities of the lacrimal ducts, deformed ears and ear canals, hyperplastic mucosa, cheilitis angularis, renal dysplasia, inguinal hernia, hypospadias in males, urethral reflux, and perioral ulcer (10, 11)
Limb-mammary (LMS syndrome, MIM 603543)	Characterized by mammary gland and/or nipple hypoplasia, lacrimal duct obstruction, cleft palate with or without bifid uvula, dystrophic nails, hypohydrosis, and teeth defects (12, 13)

There are multiple classifications for cleft hand defined in medical literature but one classified by Manske and Halikis is most commonly used. This proposed classification is based on the characteristics of the thumb web, which are more important to the function of the hand than are the central deficiency features. (14) There are five types of Split hand/foot malformation syndrome (SHFM) syndrome with different chromosomal associations and genes thought to be responsible for SHFM (Table 2) (15)

**Table 2:** **Classification of split hand/foot malformation syndrome by Manske and Halikis (14)**.

Type of SHFM	Description	Characteristic	Chromosomal location	Candidate gene
I	Normal web	Thumb web space is not narrowed	7q21	DLX5, DLX6, DSS1
IIA	Mildly narrowed web	Thumb web space is mildly narrowed	Xq26	FGF13, TONDU
IIB	Severely narrowed web	Thumb web space is severely narrowed	
III	Syndactylized web	Thumb and index rays syndactylized, web space obliterated	10q24	Dactylin, SUFU, BTRC
IV	Merged web	Index ray suppressed, thumb web space is merged with the cleft	3q27	TP63
V	Absent web	Thumb elements suppressed, ulnar rays remain, thumb web space no longer present	2q31	DLX1, DLX2

Ectrodactyly is usually seen as either complete absence of or malformation of one or more fingers or toes. Patients generally have median cleft in upper and lower limbs, which makes the affected limbs look like a lobster claws and hence the name given. This lobster claw is thought to arise as a result of a wedge-shaped defect of the apical ectoderm of the limb buds (16). Sometimes all four limbs involvement may be seen, even though this is a rare phenomenon. The majority of the patients usually have mild limb abnormality and very rarely may be unaffected. The patients of EEC may sometimes have webbing or fusion (syndactyly) of the fingers and/or toes (17). In some cases, syndactyly may be the only limb defect that is seen. Affected children may have other facial anomalies that includes cleft lip/palate, maxillary hypoplasia, long philtrum, and choanal atresia or can be normal too (18, 19).

The spectrum of dermatological manifestation associated with ectodermal dysplasia is variable and include hyper keratosis, thickened scaly skin to hypo pigmented dry skin with poor hair growth. Scalp hair as well as eyebrows may be sparse, wiry, and with hypo pigmented hair (20). Additional symptoms can include dysplastic nails and peg-shaped teeth (21). Tooth decay (dental caries) is very common clinical finding as seen in our patient and is often very severe and sometimes tooth enamel may be abnormal (22). There may be associated reduction in activity or complete absence of exocrine glands of the body including the sweat, salivary, lacrimal, and sebaceous glands (23). Abnormality of the sweat glands usually leads to heat intolerance and fever as an effect of hypohydrosis whereas absence of salivary glands can lead to xerostomia. There can also be associated abnormalities in other glands including lacrimal gland causing xerophthalmia (24) as well keratitis (25). Some individuals with EEC syndrome have developed hearing loss (26). Some individuals may have endocrinal problems like hypopituitarism and underdeveloped thymus (27). There have been few rare case reports of associated anomalies of the genitourinary system with various spectrum of malformations ranging from renal agenesis, renal stone (28) to hydronephrosis (29). Intelligence is usually preserved, however there may delay in speech development, which is due to associated hearing loss. Few long-term case report has shown their progression to Hodgkin lymphoma (30). There has been one case report of EEC syndrome associated with congenital heart disease (Ventricular septal defect with aortic regurgitation) (17). In other case report, Valenzise et al. reported the R298Q mutation of p63 gene in autosomal dominant ectodermal dysplasia associated with arrhythmogenic right ventricular cardiomyopathy (31). There has been no case report of EEC with TOF.

Management of this condition involves multidisciplinary involvement and starts with detailed evaluation with imaging technique of the affected limbs, ophthalmological evaluation, hearing assessment, renal ultrasound, 2D-echocardiography, thyroid screening, and skin biopsy. The confirmatory diagnosis is by molecular genetic testing. The confirmation of EEC syndrome can be done by molecular genetic testing for TP63 gene mutations (13). Patients who are diagnosed to have EEC on physical examination, mutational gene analysis of the TP63 gene should be the priority test and if it shows negative results then other test for diagnosing chromosomal abnormalities should be considered. Antenatal diagnosis is feasible using the molecular genetic testing and samples are obtained using chorionic villus sampling, which should be performed if there is suspicion on fetal ultrasound (32). Treatment is largely supportive and involves managing the various anomalies and involves a team of health care personal. Orthopedic management in form of limb reconstructive surgery may be considered in patients that are having functional disability such as ectrodactyly, syndactyly, cleft lip, or palate (33). If teeth are missing, artificial dentures may be necessary and oral hygiene makes an important part of management (34). Use of artificial tears and emollients may be necessary for ectodermal dysplasia for prevention of evaporative eye loss (35). Patel et al. reported 17-year-old man with EEC syndrome whose diagnosis was confirmed with genetic analysis showing the importance of genetic analysis and late presentation (17). Sharma et al. reported a newborn with lobster foot syndrome showing that any limb can be involved (36).

## Conclusion

EEC syndrome patients have various manifestation with various system involvement. The occurrence of EEC with cardiac disease is very rare and we have reported first case report of its type. Genetic analysis is the key for correct diagnosis of the EEC syndrome. Treatment involves multidisciplinary team, which takes care of associated malformations. Regular and strict follow up should be done of these patients. Prenatal counseling and genetic screening should be done for all couples who have previous EEC syndrome affected neonate.

## Conflict of Interest Statement

The authors declare that the research was conducted in the absence of any commercial or financial relationships that could be construed as a potential conflict of interest.

## Footnotes

^1^https://en.wikipedia.org/wiki/Vadoma
